# Seeing *beyond* COVID-19: understanding the impact of the pandemic on oncology, and the importance of preparedness

**DOI:** 10.1007/s40656-020-00351-w

**Published:** 2020-11-18

**Authors:** Daniele Carrieri, Fedro Alessandro Peccatori

**Affiliations:** 1 College of Medicine and Health and Wellcome Centre for Cultures and Environments of Health, University of Exeter, Exeter, UK; 2 European Institute of Oncology IRCCS, Via Ripamonti 435, 20141 Milan, Italy

**Keywords:** COVID-19, Oncology, Preparedness

## Abstract

The impact of this pandemic is not only through COVID-19 itself: the care for non-COVID-19 related conditions has been dramatically curtailed, shaking entire healthcare services around the world. Amongst the non-COVID-19 related conditions, oncology has been disproportionally affected. We discuss how oncology has changed since the acute phase of the pandemic; its impact on clinicians, trainees, and patients; and offer some medical and historical perspectives to reflect on how this impact could be reduced.

## Impact of COVID-19 on oncology

In this short commentary we wish to explore the question of the impact of COVID-19 on oncology. Our interest in this question stems from our experience and expertise in public health, and healthcare workforce training and burnout. Moreover, being one of us a practicing oncologist in Europe, oncology was chosen as a case study for its relevant impact on health care systems, and more in general on patients’ health. This commentary has many limitations—for example it will not represent the patient perspective, but it will mostly focus on our areas of expertise outlined above. Our aim is to encourage more serious thinking around an area that is becoming increasingly pressing and that deserves much more discussion.

A survey of 155 countries conducted by the World Health Organization (WHO) in May 2020 reported that the COVID-19 pandemic has curtailed the provision of health services for non-communicable diseases (Dyer 2020). Key resources, services, and healthcare staff have been re-directed towards COVID-19. Oncology care, which often involves extensive surgery, immune suppressive therapy and inpatient treatment, has been disproportionally affected (Rosenbaum 2020). Oncologists in many countries have been redeployed to help colleagues dealing with COVID-19 patients, and ‘non-essential’ tests and screenings have been cancelled or postponed (The Lancet Oncology 2020). These changes resulted in the closure of screening and diagnostic services, a slowing down or complete cessation of referrals, significant delays and difficulties in accessing chemotherapy, and, at least in some instances, the delivery of suboptimal care. Such changes were justified as a way to minimise risks of oncology patients—many of whom are considered vulnerable—contracting COVID-19 and worsening their condition (The Lancet Oncology 2020). However, these changes resulted also in a worrying decrease in cancer diagnoses, and an increase in cancer deaths (Jones et al. 2020).

Moreover, the impact of COVID-19 on oncology has hit the wider oncology community, from research and collaborations, to education and clinical training (The Lancet Oncology 2020). For example, COVID-19 has drastically impacted the way oncology is taught in Universities and at postgraduate level. In-person classes were put on hold during the acute phase of COVID-19 pandemic, to comply with social distancing measures, and protect students’ health. Hands-on teaching has been replaced by webinars and online resources. Video conference-based case discussions have been used as proxy of real case management (The Lancet Oncology 2020). Will these changes have an impact on the skills and competences of future oncologists? Academic bodies, together with student representatives, are considering a blended model of medical teaching. The use of online platforms might replace frontal lessons, but clinical experience cannot be substituted and should be gradually re-introduced, maintaining the flexibility and adaptiveness needed in this new situation.

## A slow return to ‘normality’?

Dealing with the urgent and growing oncology patient backlog after the peak of the pandemic is proving incredibly challenging.

In England alone, experts have estimated that as a direct result of not having received healthcare in recent months 30,000 to 40,000 patients could not start oncology treatment, and between 7000 and 35,000 of those could die in the next year.^1^

Some oncology patients may also still feel unsafe in hospital or primary care settings, and/or may not want to burden healthcare professionals (in the UK there have been 60% fewer referrals than usual since the pandemic (Jones et al. 2020)). Although the uptake of telemedicine may help reaching some patients with potential cancer symptoms needing urgent assessment—this and other forms of remote medicine may not be accessible to patients from low socioeconomic backgrounds, thus exacerbating inequalities in cancer diagnosis and care (Jones et al. 2020). Moreover, clinicians themselves need to acquire new skills e.g. how to use telemedicine platforms appropriately, or assessing patients who are wearing face coverings. Some oncology healthcare professionals have experienced significant burnout, as a consequence of having worked in COVID-19 hospitals, moral injury resulting from being unable to give patients the best care due limited resources, and a lack of organisational and psychological support (Greenberg et al. 2020).

In the eventuality of a second wave of clinically significant SARS-CoV-2 pandemic, a return to a COVID-19-based healthcare service will have catastrophic consequences for cancer patients and is also likely to exacerbate burnout amongst the oncology and wider healthcare workforce.^2^

## Medical perspective

It is still too early to appreciate the direct and indirect impact of COVID-19 on oncology and on healthcare more broadly. However, there are some important points that need to be considered, particularly in the event of a new COVID-19 outbreak.

From a medical—and specifically oncological—perspective there have been calls to avoid compromising the delivery of care when the risk of COVID-19 infection (and any resulting complications) is outweighed by the benefits of treatment (Spicer et al. 2020).

It is also important to highlight that the oncology patient backlog, workforce burnout, and the other issues sketched above should not solely be attributed to the recent response to COVID-19. Oncology care (like most healthcare) was already under considerable pressure before the pandemic (Carrieri et al. 2018). In many countries the increased demand for oncology care has not been followed by a rise in the oncology workforce and appropriate infrastructures (Sullivan et al. 2011). Thus, COVID-19 has exacerbated pre-existing longstanding problems in oncology care, which are in turn linked to a broader unpreparedness from a global health perspective.

## History of pandemics perspective

From a history of pandemics perspective, one of the most important issue raised by COVID-19 is the importance of preparedness. Historians of medicine coined terms such as ‘global amnesia’ to refer to a recurring pattern in human history to forget about pandemics as soon as they recede, and being unprepared once they re-emerge (Desai 2020). As Frank M. Snowden points out, one of the most significant and recent alarm bells was the report ‘A World at Risk’,^3^ published by the WHO in 2019 (one year after the beginning of a new Ebola epidemic in the Democratic Republic of Congo), which concluded that the world and its individual countries were comprehensively unprepared for long-anticipated global health threats (Snowden 2019).

Despite these warnings, the world remained significantly unprepared, and such unpreparedness is ultimately linked to the global ‘tsunami’ of COVID-19 cases, and the reactive, hurried, and catastrophic curtailing of most oncology care to tackle the peak of the COVID-19 pandemic. Complex global threats like COVID-19 require complex system approaches, which must comprise appropriate prevention measures. We need more proactive strategies based on investments in sustainable public and global health, including the regular assessment of the likely global challenges and the development of means to meet them; investment in scientific research; enhanced healthcare infrastructure; and investments promoting close international collaboration, innovative clinical training models, and health education (Snowden 2019).

## Data Availability

N/A.

## Footnotes

1 https://www.theguardian.com/world/2020/aug/06/nhs-shutdown-risks-thousands-of-deaths-in-covid-19-second-wave (last accessed 07/09/2020).

2 https://www.theguardian.com/society/2020/sep/05/more-than-1000-doctors-want-to-quit-nhs-over-handling-of-pandemic?CMP = Share_AndroidApp_Other (last accessed 07/09/2020).

3 https://apps.who.int/gpmb/assets/annual_report/GPMB_annualreport_2019.pdf (last accessed 14/09/2020).
